# Microglial heterogeneity and complement component 3 elimination within emerging multisensory midbrain compartments during an early critical period

**DOI:** 10.3389/fnins.2022.1072667

**Published:** 2023-01-04

**Authors:** Julianne B. Carroll, Shaida Hamidi, Mark L. Gabriele

**Affiliations:** Department of Biology, James Madison University, Harrisonburg, VA, United States

**Keywords:** inferior colliculus, multisensory, C3, CR3/CD11b, development, refinement, pruning

## Abstract

The lateral cortex of the inferior colliculus (LCIC) is a midbrain shell region that receives multimodal inputs that target discrete zones of its compartmental (modular-matrix) framework. This arrangement emerges perinatally in mice (postnatal day, P0-P12) as somatosensory and auditory inputs segregate into their respective modular and matrix terminal patterns. Microglial cells (MGCs) perform a variety of critical functions in the developing brain, among them identifying areas of active circuit assembly and selectively pruning exuberant or underutilized connections. Recent evidence in other brain structures suggest considerable MGC heterogeneity across the lifespan, particularly during established developmental critical periods. The present study examines the potential involvement of classical complement cascade signaling (C3-CR3/CD11b) in refining early multisensory networks, and identifies several microglial subsets exhibiting distinct molecular signatures within the nascent LCIC. Immunostaining was performed in GAD67-green fluorescent protein (GFP) and CX3CR1-GFP mice throughout and after the defined LCIC critical period. GAD labeling highlights the emerging LCIC modularity, while CX3CR1 labeling depicts MGCs expressing the fractalkine receptor. C3 expression is widespread throughout the LCIC neuropil early on, prior to its conspicuous absence from modular zones at P8, and more global disappearance following critical period closure. CD11b-expressing microglia while homogeneously distributed at birth, are biased to modular fields at P8 and then the surrounding matrix by P12. Temporal and spatial matching of the disappearance of C3 by LCIC compartment (i.e., modules then matrix) with CD11b-positive MGC occupancy implicates complement signaling in the selective refinement of early LCIC connectivity. Multiple-labeling studies for a variety of established MGC markers (CD11b, CX3CR1, Iba1, TMEM119) indicate significant MGC heterogeneity in the LCIC as its compartments and segregated multisensory maps emerge. Marker colocalization was the exception rather than the rule, suggesting that unique MGC subpopulations exist in the LCIC and perhaps serve distinct developmental roles. Potential mechanisms whereby microglia sculpt early multisensory LCIC maps and how such activity/inactivity may underlie certain neurodevelopmental conditions, including autism spectrum disorder and schizophrenia, are discussed.

## Introduction

Synaptic pruning is an essential component of neurodevelopment that involves selective removal of extraneous or underutilized contacts (Wilton et al., 2019). Microglial cells (MGCs), the resident macrophages of the nervous system, play a pivotal role in this process of network refinement during early critical periods (Paolicelli et al., 2011; Hong et al., 2016; Mosser et al., 2017; Thion and Garel, 2017, 2020). MGCs exhibit various activation states, from highly phagocytic during periods of targeted engulfment of opsonized material, to resting or surveilling states after circuits are established and functionally-tuned (Jurga et al., 2020). Aberrations in MGC pruning behaviors are thought to underlie certain neurodevelopmental conditions (Zhan et al., 2014; Bordeleau et al., 2019; Faust et al., 2021), including autism spectrum disorders (ASD, under-pruning) and schizophrenia (over-pruning).

Recent single-cell RNA sequencing studies reveal microglial heterogeneities throughout the brain, with the most significant diversity exhibited during developmental critical periods (De Biase et al., 2017; Hammond et al., 2019; Li et al., 2019; Masuda et al., 2019, 2020; Tan et al., 2020). Given the differential expression of MGC transcriptional markers observed across the lifespan, various conditions and brain regions, it is reasonable that distinct microglial subsets serve timely roles with unique responsibilities. Two canonical developmental MGC gene clusters involve the expression of fractalkine and classical complement signaling cascade components, each of which include a host of specific markers.

The fractalkine pathway, most typically implicated in microglial recruitment and migration, involves interactions of the neuronally-expressed fractalkine ligand (CX3CL1) with its corresponding receptor (CX3CR1) found exclusively on MGCs (Harrison et al., 1998; Jung et al., 2000; Paolicelli et al., 2014; Arnoux and Audinat, 2015). CX3CL1 can either be membrane-bound, or liberated when cleaved by the metalloproteinase ADAM10 (Gunner et al., 2019). MGC occupancy along with aspects of synaptic pruning/maturation are delayed in developing barrel fields (Hoshiko et al., 2012) and hippocampus (Paolicelli et al., 2011; Pagani et al., 2015) in CX3CR1 mutants, suggesting involvement of MGCs and fractalkine signaling in governing important features of early circuit assembly.

The classical complement cascade, in particular complement component 3 (C3) and its receptor CR3, appear equally involved in MGC-neuronal crosstalk during development and certain disease states (Stevens et al., 2007; Schafer et al., 2012; Stephan et al., 2012). An initiating protein, C1q, produced by MGCs attaches to cells or portions of cells marking them for subsequent removal. C1q tagging ultimately leads to the production of the opsonin C3. MGCs initiate phagocytosis of selectively tagged debris *via* C3 binding with its cognate receptor, complement receptor 3 (CR3) (Stephan et al., 2012). CR3 is a heterodimer of integrin αM (CD11b) and β2 (CD18) subunits, with CD11b being an established marker for CR3-expressing MGCs (Bajic et al., 2013; Vorup-Jensen and Jensen, 2018; Lamers et al., 2021).

To explore potential microglial influences on the assembly of multisensory circuits, the present study focuses on a specific shell region of the midbrain inferior colliculus (IC). The IC consists of three subdivisions; the central nucleus (CNIC), the dorsal cortex (DCIC), and the lateral cortex (LCIC). Though classically described as an auditory relay hub due to its well-documented CNIC, the LCIC exhibits multimodal response properties (Aitkin et al., 1978, 1981; Jain and Shore, 2006; Gruters and Groh, 2012) that emerge from its discretely-mapped, network configuration. In adult mice, somatosensory projections target patchy zones positive for glutamic acid decarboxylase (GAD), termed modules, while auditory inputs terminate throughout the encompassing matrix (Lesicko et al., 2016, 2020). Discontinuous LCIC modular fields span its intermediate layer (layer 2, Chernock et al., 2004) and are completely surrounded by a calretinin-positive matrix (layers 1 and 3; Dillingham et al., 2017; Gay et al., 2018). These and other identified neurochemical and guidance markers reliably label the emerging LCIC compartmental structure and were instrumental in defining an early critical period regarding its characteristic cytoarchitectural organization and interfacing multisensory afferent patterns (from birth to postnatal day 12, P12; Dillingham et al., 2017; Gay et al., 2018; Stinson et al., 2021). Inputs of somatosensory and auditory origin exhibit considerable overlap initially, prior to fully segregating into modality-specific compartments (modular and matrix, respectively; Lamb-Echegaray et al., 2019; Weakley et al., 2022).

Recently, we reported that microglial colonization of the LCIC and timely occupancy of its emerging modules by CX3CR1-positive MGCs is fractalkine signaling-dependent (Brett et al., 2022). The present study explores the potential role complement signaling may play in refining early LCIC connectivity. Here, developmentally regulated changes in C3 and CD11b expression with respect to emerging LCIC compartments are documented in a series of GAD67-green fluorescent protein (GFP) mice. Additionally, we examine the possibility of molecularly distinct MGC subpopulations in the neonatal LCIC by concurrently labeling for several established MGC markers (CD11b, TMEM119, Iba1) in CX3CR1-GFP mice. The results reveal widespread C3 expression that is subsequently pruned in a compartmental-specific progression (i.e., modules first, then matrix), and that such refinement appears temporally and spatially linked to the presence of CD11b-expressing MGCs. Examined MGC markers were generally non-overlapping, providing evidence for the likelihood of considerable MGC heterogeneity in the developing LCIC.

## Materials and methods

### Animals

A developmental series of mice spanning the established LCIC critical period (P0, P4, P8, P12; *n* ≥ 3 at each age) of two transgenic lines [GAD67-GFP (*n* = 46) and CX3CR1-GFP (*n* = 47)] were used for experimentation. In some instances, an additional developmental timepoint was examined well after critical period closure (P36). GAD67-GFP knock-in mice enabled easy identification of emerging LCIC modules, and thus assessments of various MGC markers in relation to developing LCIC compartments. Previous efforts from our lab verifying the specificity of this mouse line are published elsewhere (Gay et al., 2018). Details regarding the targeting of enhanced GFP to the locus of the GAD gene, breeding strategies, and genotyping sequences have been previously described (Yanagawa et al., 1997; Tamamaki et al., 2003; Ono et al., 2005). Breeding pairs for this line were originally furnished by Dr. Peter Brunjes (University of Virginia School of Medicine) with permission granted from Dr. Yuchio Yanagawa (Gunma University Graduate School of Medicine, Gunma, Japan). CX3CR1-GFP mice (JAX 005582) facilitated visualization of fractalkine-receptor expressing MGCs in heterozygous and homozygous progeny. Similar to wild-type, CX3CR1^+/GFP^ mice are capable of fractalkine signaling, whereas such functionality is compromised in CX3CR1*^GFP/GFP^* littermates. In the present study, only heterozygous mice for this line were used and are referred to simply as CX3CR1-GFP hereafter. CX3CR1-GFP mice were used to assess potential MGC heterogeneity and relative colocalization with a variety of other MGC markers. Previous work in our lab confirmed the presence of CX3CR1-expressing MGCs in the nascent LCIC and characterized migratory behaviors that recognize its emerging compartments (Brett et al., 2022) and temporally correlate with peak shaping of its multisensory afferent streams (Lamb-Echegaray et al., 2019; Weakley et al., 2022).

Both strains were bred on a C57BL/6J background (JAX 000664). GAD-GFP breeding consisted of pairing heterozygous males with C57BL/6J females. Progeny were observed with a Dark Reader Spot Lamp and viewing goggles prior to P4 to identify GFP-expressing pups (Clare Chemical Research, Dolores, CO, USA, Cat# SL10S)^1^. CX3CR1-GFP genotyping was outsourced (Transnetyx, Cordova, TN, USA)^2^ using probes specific for this JAX line from the provided database. Approximately equal numbers of male and female subjects were used for all experimentation, and no sex-specific differences were observed. All experimental procedures are in accordance with National Institutes of Health guidelines and have been approved by the IACUC of James Madison University (No. 20-1421).

### Perfusions and tissue sectioning

In preparation for midbrain sectioning, mice were given an overdose of ketamine (200 mg/kg) and xylazine (20 mg/kg). Brains were transcardially perfused with physiological rinse (0.9% NaCl and 0.5% NaNO_2_ in dH_2_O) followed by 4% paraformaldehyde, and then by a solution of 4% paraformaldehyde and 10% sucrose for cryoprotection. Brains were removed from the skull and stored at 4^°^C in 4% paraformaldehyde/10% sucrose solution for 24 h, and then were allowed to equilibrate at the same temperature in a solution of 4% paraformaldehyde/30% sucrose. The tissue block was cut in the coronal plane at 50 μm using a sliding freezing microtome and sections throughout the rostrocaudal extent of the midbrain were collected in 0.1 M phosphate-buffered saline (PBS, pH 7.4).

### Immunocytochemistry

To begin processing for various markers of interest (see Table 1 for all relevant antibody information), sections were rinsed in phosphate buffered saline (PBS) three times for five minutes each. Tissue sections were then blocked in 5% normal serum for 30 min (species of normal serum always corresponded to species of secondary antibody production). Blocking was followed immediately by transfer to primary antibody solution at the identified optimal dilution and sections were agitated overnight at 4^°^C.

**TABLE 1:** Antibody information.

Antibody name	Structure of immunogen	Manufacturer info	Concentration used
Anti-C3	Lyophilized powder of IgG fraction to mouse complement C3 and buffer salts	MP Biomedicals, 0855463, goat, RRID:AB_2334481	1:200
Anti-CD11b, clone 5C6	Purified IgG prepared by affinity chromatography on protein G from tissue culture supernatant	Bio-Rad, MCA711G, rat, monoclonal, RRID:AB_323167	1:200
Anti-Iba1	Synthetic peptide that corresponds to the C-terminus of Iba1	Wako Chemicals, 019-19741, rabbit, polyclonal, RRID:AB_839504	1:1000
Anti-TMEM119	Recombinant fragment (GST-tag) within Mouse TMEM119 aa 100 to the C-terminus (intracellular)	Abcam, AB209064, rabbit, monoclonal RRID:AB_2800343	1:150
Alexa Fluor 350 goat anti-rat IgG	IgG recognizes both heavy and light chains from rat	Thermo Fisher Scientific, A21093, RRID:AB_2535748	1:25
Biotinylated horse anti-rabbit IgG	IgG recognizes both heavy and light chains from rabbit	Vector Laboratories, BA-1100, RRID:Ab_2336201	1:600
Biotinylated horse anti-goat IgG	IgG recognizes both heavy and light chains from goat	Vector Laboratories, BA-9500, RRID:AB_2336123	1:600
Biotinylated goat anti-rat IgG	IgG recognizes both heavy and light chains from rat	Vector Laboratories. BA-9401, RRID:AB_2336208	1:600

For double-immunolabeling experiments, sections were allowed to equilibrate to room temperature the following day for 20 min before rinsing in PBS three times for eight minutes each. Tissue was then incubated for 90 min in a directly-conjugated secondary antibody (Alexa Fluor 350). After an additional four PBS rinses of 10 min each, tissue was blocked in the appropriate 5% normal serum prior to incubation in the second primary antibody at 4^°^C overnight.

On the final day of processing, tissue was once again allowed to return to room temperature, then rinsed three times in PBS. Next, an appropriate biotinylated secondary antibody was applied for 1 h, followed by three additional PBS rinses. Lastly, a streptavidin fluorescent conjugate (DyLight 549 streptavidin, 1:200, Vector Laboratories, Newark, CA, USA, SA-5549, RRID:AB_2336408) was applied for 2 h before three final PBS rinses. Tissue was then mounted onto slides and coverslipped with Prolong Diamond anti-fade mountant (Thermo Fisher Scientific, Waltham, MA, USA, P36970). For single-labeling studies, the biotinylated amplification described above was used in lieu of a directly conjugated secondary approach. For TMEM119 processing, tissue was blocked in 10% instead of 5% normal serum, and all steps beginning with the initial PBS rinses through the biotinylated secondary included 0.5% Triton X-100 (Millipore Sigma, Burlington, MA, USA, TX1568).

### Epifluorescent imaging

Wide-field image capturing was performed on a Nikon Eclipse Ti-2 microscope (Nikon, Melville, NY) using a monochrome, Hamamatsu ORCA-Flash 4.0 V3 CMOS camera (Hamamatsu, Bridgewater, NJ) and PlanApo objectives (10x, 20x, 40x; NA = 0.30, 0.75, and 1.30, respectively). Filter sets (Chroma Technology, Bellows Falls, VT) were designed with careful attention to the spectra of the various fluorophores to make certain no bleed through exists between channels. An extended depth of field (EDF) algorithm was used to generate two-dimensional images from acquired Z-stacks (Elements Software; Nikon). The Nikon Elements EDF module facilitates merging of captured Z-stacks into two-dimensional images, using only the focused regions for each optical plane. Separate channels were pseudocolored and lookup tables were adjusted slightly for each image to reduce background noise and best depict labeling observed through the microscope. Images were saved as lossless JPEG2000 files prior to subsequent quantitative analyses. Masking of minimal artifact observed outside section contours in a few instances was performed (Adobe Photoshop, San Jose, CA) to enhance overall figure quality.

### LCIC modular identification, C3 brightness profile sampling, and areal coverage assessments

Raw JPEG2000 images were opened in NIS-Elements and exported as uncompressed TIFF files and imported into ImageJ software (NIH, Bethesda, MD, RRID:SCR_003070)^3^ in order to generate brightness plot profiles for C3 and GAD channels at P4, P8, and P12. Mid-rostrocaudal sections of the IC where LCIC modularity was most apparent were used. Sampling focused on these ages as qualitative C3 observations appeared to progress from the earliest indications of C3 disappearance (P4), to strikingly discontinuous C3 patterns (P8), to absence of discernible C3 labeling (P12). Merged channels were first separated and converted to grayscale, and then a threshold function was performed to facilitate clear delineation of modular boundaries (for more information see Brett et al., 2022). A freehand tool (line thickness of 100) was used to sample and fully encompass layer 2 GAD-positive modules in a ventral-to-dorsal progression. Once the sampling contour was set, a region of interest (ROI) function was utilized to duplicate and save the exact sampling contour for use in the other channel of the same image. Sampling data for the channels provided brightness profile patterns of the fluorescent markers with respect to one another.

For C3 and CD11b areal coverage comparisons at critical stages (i.e., P4 and P8 for C3, and P8 and P12 for CD11b), extracted GAD and C3 or CD11b channels from 20x digital merges were opened in FIJI (Schindelin et al., 2012). Five sections were taken from three animals for a total of fifteen sections analyzed at each age. In the GAD channel, modular boundaries were identified (Brett et al., 2022), traced with the freehand tool, and combined using the ROI manager. GAD channels were then closed and all subsequent steps were performed exclusively on corresponding C3 or CD11b channels. The entirety of the LCIC in the field of view was outlined next and added to the ROI manager. Selecting both the GAD-defined modular and total LCIC ROIs, the XOR (i.e., exclusive or operator) function was used to create a ROI for just the encompassing matrix. Additionally, a sufficiently large box was drawn in the region of the C3 or CD11b channel containing no tissue to provide a background level. Modular, matrix, and background medians for each of the ROIs were calculated. The following ratio from those median values was used to assess relative areal C3 or CD11b coverage: (module–background)/(matrix–background). Values above one correlate closely to largely modular expression, those near one indicate comparable or homogeneous compartmental expression, while those diminishing below one indicate expression preference for the surrounding matrix.

## Results

### C3 expression with respect to emerging LCIC compartments

**FIGURE 1:**
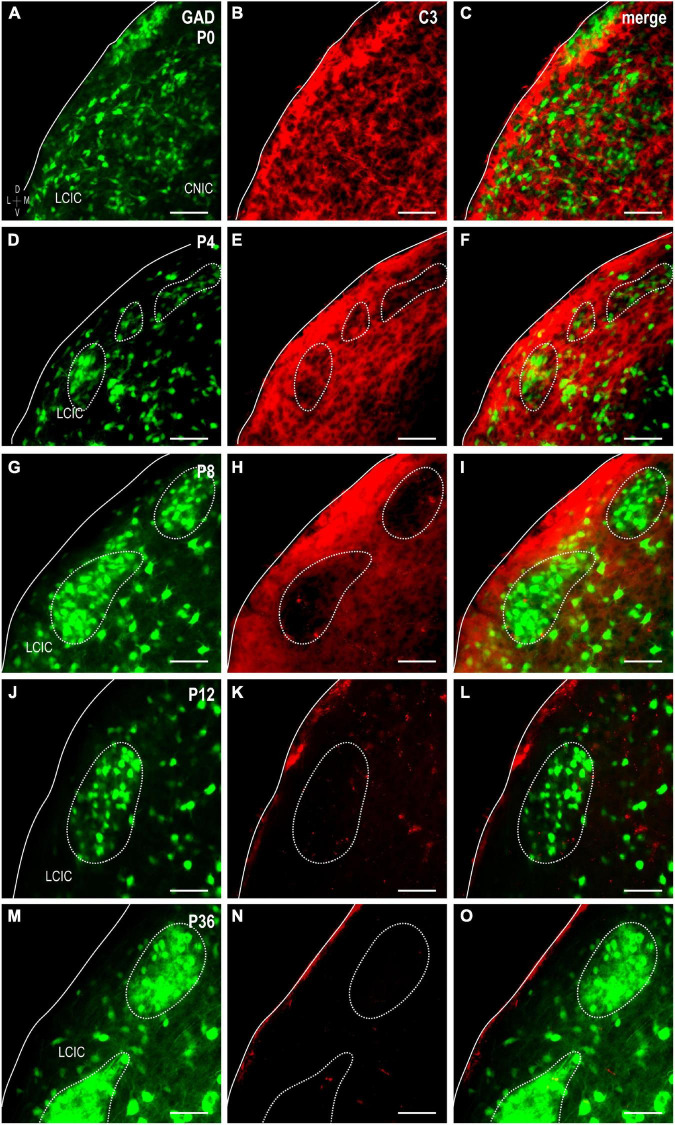
Complement component 3 (C3) expression (red) in a developmental series [P0, **(A–C)**; P4, **(D–F)**; P8, **(G–I)**; P12, **(J–L)**; P36, **(M–O)**] of glutamic acid decarboxylase (GAD)-green fluorescent protein (GFP) (green) mice. Hints of GAD-positive lateral cortex of the inferior colliculus (LCIC) modules emerge by P4 and become increasingly apparent with age (*dashed contours*). A diffuse plexus of C3 neuropil expression dominates the LCIC at birth **(B,C)**, with many of its voids occupied by GAD-positive somata. As LCIC modules begin to organize at P4, the slightest indication of C3 loss is observed within these domains **(E,F)**. By P8, C3 expression is completely lacking within GAD-defined modular zones, yet remains strongly concentrated throughout the surrounding matrix **(H,I)**. C3 expression is gone from both LCIC compartments at P12 coincident with its critical period closure **(K,L)**, and remains so thereafter [P36; **(N,O)**]. Scale bars = 50 μm.

To explore the potential involvement of the complement system on the refinement of segregating LCIC multisensory maps (Weakley et al., 2022), C3 expression was examined in a developmental series of GAD67-GFP mice (Figure 1). Timepoints included four equally spaced stages spanning its established critical period (Figures 1A–L), as well as one well after its closure (Figures 1M–O). C3 expression is strong at birth, dominating the neuropil throughout the LCIC (Figures 1A–C). Many of the small lacunae observed in the dense plexus of C3 immunostaining were occupied by GAD-positive cells (Figure 1C). Coincident with the first hints of primitive GAD cell clusters at P4 was the earliest indication of similarly patterned and seemingly spatially matched C3 loss (Figures 1E, F). By P8, conspicuous intermittent C3-negative patches were now readily apparent within an encompassing plexus of C3-positive matrix (Figures 1G–I). Discontinuous LCIC patches lacking C3 expression reliably aligned with the well-established GAD-positive modular zones at this age (Figure 1I). LCIC C3 expression tails off by the critical period end at P12 (Figures 1J–L) once multimodal afferents have fully segregated. Absence of any noteworthy C3 was consistently observed at P36, confirming the transient nature of its early postnatal expression (Figures 1M–O).

### C3 quantification at pivotal LCIC timepoints and areal coverage

**FIGURE 2:**
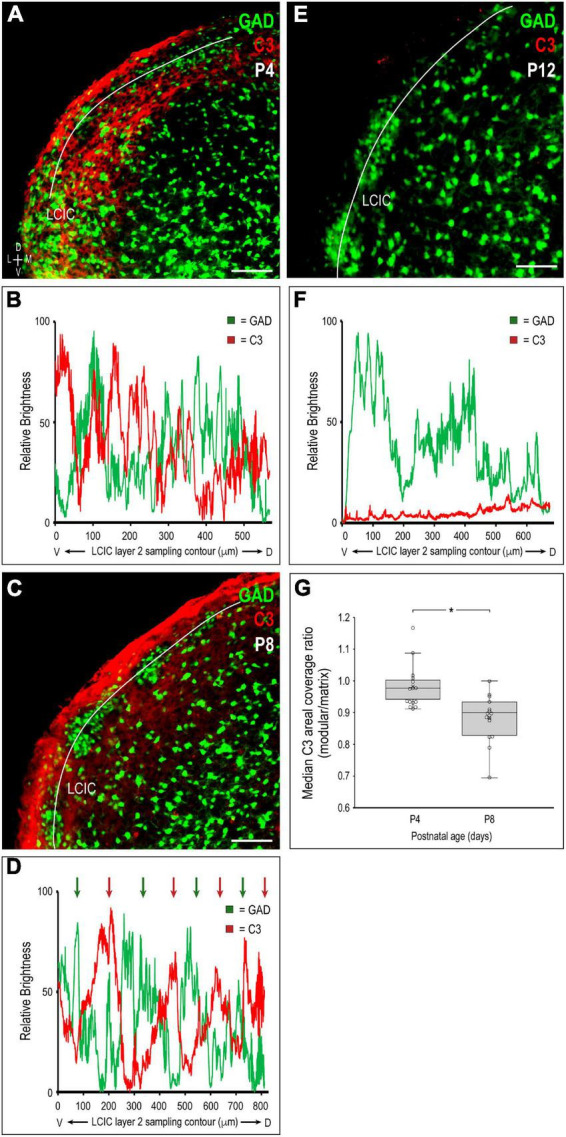
Quantification of complement component 3 (C3) expression patterns with respect to the emerging lateral cortex of the inferior colliculus (LCIC) compartmental structure. Multichannel region of interest (ROI) contour sampling of LCIC layer 2 modular fields at P4 **(A)**, P8 **(C)**, and P12 **(E)** with corresponding glutamic acid decarboxylase (GAD) (green) and C3 (red) expression profiles **(B,D,F)**. Although C3 signal fluctuations are evident at P4, they still appear largely unorganized with respect to emerging GAD-positive modules **(B)**. By P8, clear periodicities are observed for both channels with waveforms that are consistently out-of-phase with each other [*green and red arrows* in **(D)**]. Such spatial offset confirms modular-specific C3 loss at this age, with strong expression persisting throughout the encompassing matrix. By LCIC critical period closure at P12, C3 expression is barely detectable **(F)**, suggesting its clearance from both LCIC compartments. Box and whisker plots comparing median compartmental areal coverage ratios at critical period stages P4 and P8 **(G)**. Their vertical separation demonstrates significant differences in modular C3 expression at these ages (*p < 0.001). Note that the disjoint P4 and P8 boxes indicate that 3/4 of the P4 ratios are above 3/4 of the P8 ratios. P4 values clustered about or just below 1 suggest a transition at this age from homogeneous C3 LCIC expression seen earlier, to the beginnings of selective C3 disappearance within modular confines. Markedly lower P8 values quantitatively indicate continued modular-specific C3 loss. Horizontal lines within boxes represent medians; X’s within boxes represent means. Scale bars in **(A,C,E)** = 100 μm.

Representative brightness plot profiles generated from LCIC layer 2 sampling at P4 and P8, and P12 confirm observed changes in C3 expression with respect to developing LCIC compartments (Figures 2A–F). Channel-specific waveforms at P4 (Figures 2A, B) reveal hints of emerging periodic signal fluctuations in C3 expression (red) relative to the still developing LCIC modularity (green). At P8 (Figures 2C, D), consistently out-of-phase waveforms for C3 and GAD are reliably observed, highlighting the fact that C3 voids (i.e., troughs in red signal) precisely align with clearly defined GAD-positive modules (i.e., peaks in green signal). Lack of noteworthy C3 signal at P12 suggests its downregulation or clearance from both LCIC compartments by its critical period closure (Figures 2E, F). Median LCIC areal intensity measures (Figure 2G) support these qualitative observations. Calculated compartmental C3 ratios (modules/matrix) decrease significantly from P4 to P8 (*p* < 0.001), as C3 modular voids become increasingly apparent (i.e., matrix-only expression by P8).

### Spatiotemporal compartmental localization of CD11b-positive MGCs

**FIGURE 3:**
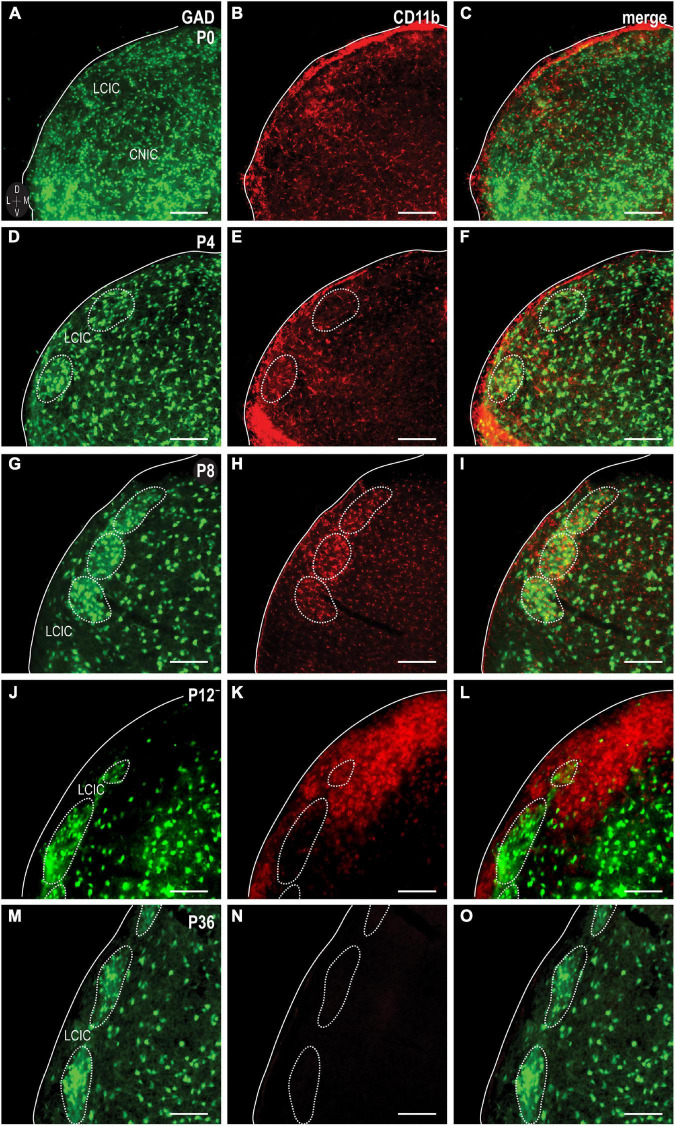
Integrin αM (CD11b) expression (red) in a developmental series [P0, **(A–C)**; P4, **(D–F)**; P8, **(G–I)**; P12, **(J–L)**; P36, **(M–O)**] of glutamic acid decarboxylase (GAD)-GFP (green) mice. CD11b expression is apparent and homogeneously distributed throughout IC subdivisions at birth, prior to concentrating more toward the lateral cortex of the inferior colliculus (LCIC) by P4. At P8, CD11b expression localizes most notably within LCIC GAD-defined modular fields. CD11b-expressing microglial cells (MGCs) shift to occupying the surrounding matrix at P12. By P36, CD11b expression is largely undetectable in the LCIC. Scale bars = 100 μm.

**FIGURE 4:**
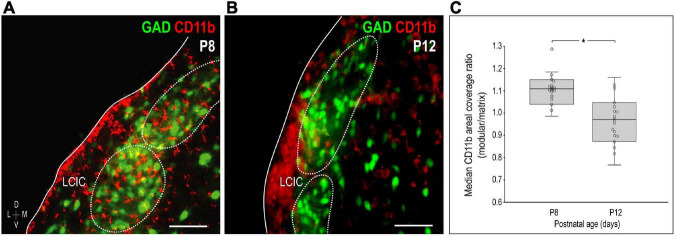
Changing compartment-specific integrin αM (CD11b) patterns from P8 to P12. CD11b expression (red) at P8 is concentrated in the lateral cortex of the inferior colliculus (LCIC) and most concentrated within glutamic acid decarboxylase (GAD)-positive modules [green, **(A)**]. CD11b expression at P12 is complementary to that observed at P8, with labeling that now predominates in the surrounding matrix **(B)**. Areal coverage analyses for CD11b **(C)** support qualitative observations, showing significant shifts in CD11b compartmental expression from P8 (primarily modular) to P12 (primarily matrix, *p < 0.01). Scale bars in **(A,B)** = 50 μm.

Similar to the above-mentioned C3 experiments, CR3/CD11b-expressing microglia were examined relative to the developing LCIC modular-matrix framework (Figure 3). CD11b expressing MGCs colonize all three IC subdivisions at birth (Figures 3A–C). Starting at P4, CD11b becomes noticeably more restricted to the LCIC as compared to the neighboring CNIC (Figures 3D–F). By P8, CD11b-positive microglia are even more biased to the LCIC and most heavily concentrated within layer 2, overlapping GAD-defined modular fields (Figures 3G–I). A largely complementary pattern is seen at P12 with MGC location now preferential for the surrounding matrix, suggesting that complement signaling microglia first concentrate within modular zones prior to selective occupancy of the encompassing matrix (Figures 3J–L). By P36, similar to C3 expression, CD11b-positive microglia are absent throughout the LCIC (Figures 3M–O). Higher magnification imaging highlights preferential clustering within modular confines at P8 (Figure 4A) that shifts to the surrounding matrix by P12 (Figure 4B). Median LCIC CD11b areal intensity measures (Figure 4C) yield ratios at P8 generally above one (i.e. primarily modular) that decrease significantly to generally less than one by P12 (primarily matrix, *p* < 0.01). Taken together, compartment-specific C3 disappearance (first modules, then matrix) follows similarly timed and spatially-matched aggregations of CD11b-expressing microglia. As anticipated, no co-localization between CD11b (microglial) and GAD (neuronal) labeling was observed.

### LCIC C3 clearance follows shift in CD11b compartmental expression

**FIGURE 5:**
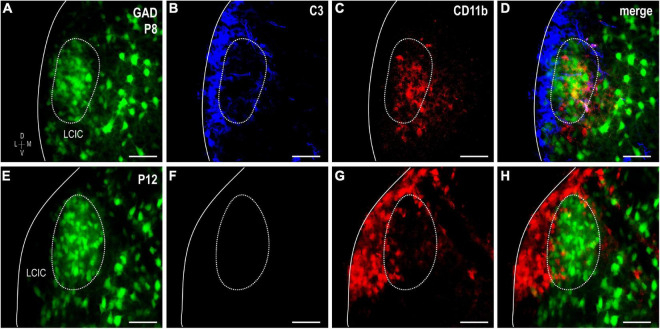
Sequential complement component 3 (C3) compartmental clearance follows shift in integrin αM (CD11b)-expressing microglial cell (MGC) location. Double-labeling studies for C3 (blue) and CD11b (red) in glutamic acid decarboxylase (GAD)-GFP (green) mice at P8 **(A–D)** and P12 **(E–H)**. At P8, CD11b-positive microglia occupy GAD-defined modules, where C3 labeling is now weak compared to the surrounding matrix. By P12, CD11b-positive MGCs preferentially congregate in the matrix where C3 expression is now also unremarkable. Scale bars = 50 μm.

Complement component 3 and CD11b were examined together in GAD-67 GFP mice at P8 and P12 to confirm that CD11b-positive MGCs concentrate within GAD modules where C3 expression initially disappears, prior to a similar progression observed in the adjacent matrix (Figure 5). Double-labeling results in GAD-GFP mice reliably show changing CD11b microglial patterns that correlate with age-matched compartmental C3 disappearance, first within layer 2 modules (P8, Figures 5A–D) followed next by the surrounding matrix (P12, Figures 5E–H).

### Evidence of LCIC microglial heterogeneity: CX3CR1 and CD11b

**FIGURE 6:**
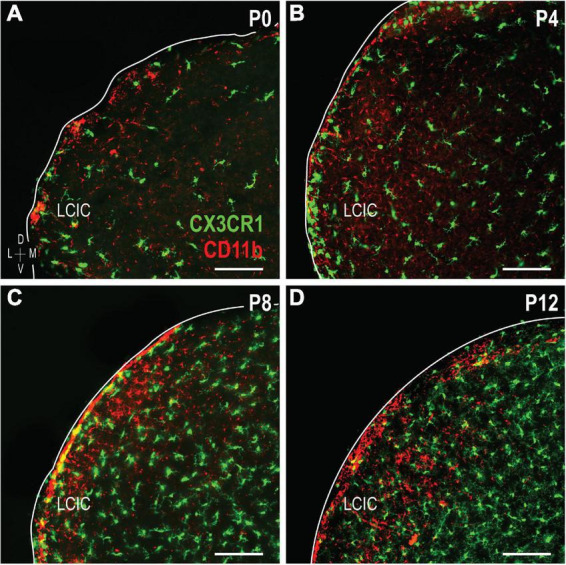
Developmental progression of digital merges of integrin αM (CD11b) immunostaining in CX3CR1-GFP heterozygous mice **(A–D)**. Microglial cells (MGCs) expressing the fractalkine receptor (CX3CR1, green) appear to be distinct subpopulations from those expressing CD11b (red) in the lateral cortex of the inferior colliculus (LCIC) throughout its early critical period. CX3CR1 patterns shown here are in keeping with those recently reported Brett et al. (2022), namely with matrix dominated expression that rings modular domains at P4/P8, prior to entering modules at P12. This progression contrasts that for CD11b, which first shows preference for LCIC modules, prior to a later shift into surrounding matrix regions. Scale bars = 100 μm.

**FIGURE 7:**
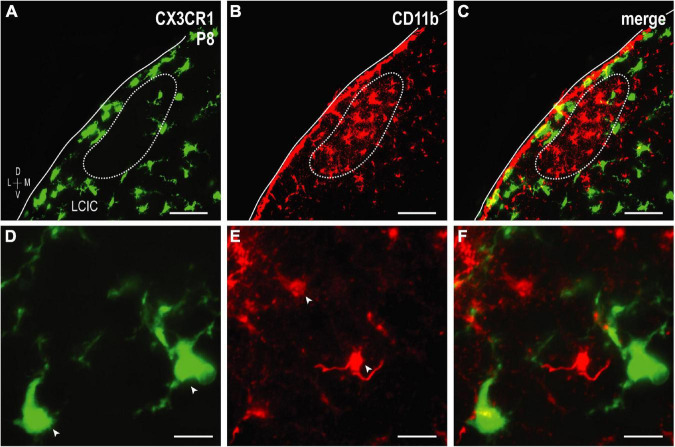
Higher magnification series of integrin αM (CD11b) expression in P8 fractalkine receptor (CX3CR1)-GFP tissue showing distinct microglial cell (MGC) subsets. CX3CR1-expressing microglia (green) at P8 surround modules [*dashed contours*, **(A–C)**, for more see Brett et al., 2022] where complement receptor expression (CD11b, red) is most concentrated. At every examined timepoint with notable CD11b expression (i.e., not P36), lateral cortex of the inferior colliculus (LCIC) CX3CR1 **(D)** and CD11b **(E)** expression was non-overlapping **(F)**, providing evidence that supports the notion of different MGC populations with unique molecular signatures. Arrowheads in **(D,E)** indicate unique microglial subpopulations. Scale bars in **(A–C)** = 50 μm; **(D–F)** = 20 μm.

The colonization and spatial patterning of fractalkine receptor expressing MGCs (CX3CR1-GFP) in the nascent mouse LCIC has recently been reported by our lab (Brett et al., 2022). Observed CX3CR1 patterns that appeared different from those described here for CD11b prompted further investigations to determine whether distinct MGC subpopulations exist in the LCIC during its early critical period. While present throughout the LCIC at birth, CX3CR1- and CD11b-positive MGCs show little to no noticeable organization and no co-localization (Figure 6A). From P4 to P8, CX3CR1-positive microglia dominate the matrix and ring modular domains that conspicuously lack fractalkine receptor expressing microglia, consistent with that described previously (Brett et al., 2022). CX3CR1 and CD11b continue to label separate MGC subpopulations, with overall spatial patterns that appear equally distinct (matrix vs. modular, respectively; Figures 6B, C). By P12, CX3CR1-positive microglia occupy LCIC modules (delayed in homozygous mice, see Brett et al., 2022), as CD11b-expressing microglia vacate said zones for the encompassing matrix (Figure 6D). Intriguingly, as LCIC modularity emerges from P4 to P12, both populations appear to have changing preferences for different aspects of the developing compartmental framework. CX3CR1-positive microglia appear to congregate in the matrix prior to invasion of the modules, and vice versa for CD11b (Figures 7A–C). Distinct subpopulations for these two microglial markers is best appreciated at higher magnification where lack of any overlap was consistently seen (Figures 7D–F).

### Further evidence of LCIC microglial heterogeneity: TMEM119 and Iba1

**FIGURE 8:**
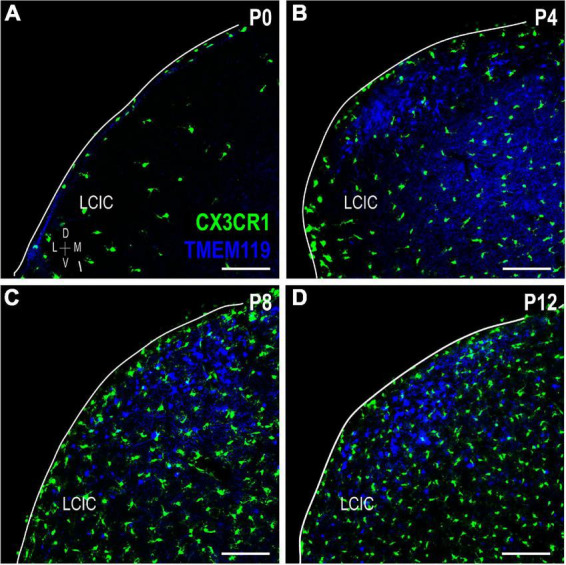
Transmembrane protein (TMEM119) expression (blue) in a developmental series **(A–D)** of fractalkine receptor (CX3CR1)-GFP (green) mice. Lateral cortex of the inferior colliculus (LCIC) TMEM119 expression is absent at birth (a) and then largely localized to layer 2 after its appearance at P4 **(B–D)**. Aside from rare double-labeled cells seen at P12 (*data not shown*), no co-localization was observed. Scale bars = 100 μm.

Lack of CX3CR1 and CD11b colocalization prompted further experimentation utilizing other established microglial markers: TMEM119 and Iba1. TMEM119 is reported to be a highly specific MGC marker in adult mouse, although there is currently no known function for the transmembrane protein (Bennett et al., 2016). TMEM119 immunocytochemistry in a developmental series of CX3CR1-GFP mice (Figure 8) shows that while absent at birth, it is detectable thereafter throughout IC, and biased toward LCIC layer 2 modular fields. Again, no significant colocalization was observed at any stage, with the only exception being the very occasional double-labeled cell encountered at P12.

**FIGURE 9:**
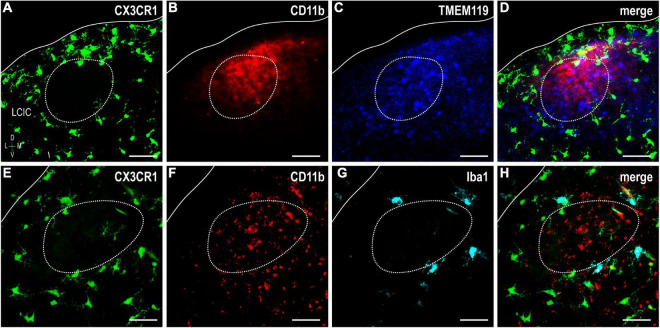
Fractalkine receptor (CX3CR1)-GFP (green), integrin αM (CD11b) (red), and transmembrane protein (TMEM119) (blue) or Iba1 (cyan) expression at P8. Significant lack of co-localization of established MGC markers supports considerable lateral cortex of the inferior colliculus (LCIC) microglial diversity during its early critical period. CX3CR1-positive microglial cells (MGCs) occupy the matrix and surround modules (*dashed contours*, for more see Brett et al., 2022) while CD11b and TMEM119 labeling exhibits a complementary modular bias **(A–D)**. Like CX3CR1, Iba1-positive microglia ring LCIC modules at P8, and are distinct from CD11b staining **(E–H)**. While all Iba1-positive cells also express CX3CR1, not all fractalkine receptor expressing cells are positive Iba1. Scale bars = 50 μm.

To confirm the relative spatial and cellular patterns observed for the various MGC markers in the same tissue, immunolabeling for both CD11b and TMEM119 was performed in P8 CX3CR1-GFP tissue (Figure 9). This timepoint was chosen for further investigation as each marker exhibits LCIC compartmental-specific expression at this age. CX3CR1-positive MGCs occupy the matrix (Figure 9A) and ring modules (for more *see* Brett et al., 2022) largely positive for CD11b- and TMEM119-expressing microglia (Figures 9B, C). Despite the similar overall preference of CD11b- and TMEM119-positive cells for modular zones at P8, little cellular colocalization was observed (Figure 9D), suggesting the presence of multiple distinct MGC subsets that may serve potentially different roles in development.

Iba1 (ionized calcium-binding adapter molecule 1), yet another MGC-specific marker, was investigated at P8 in CX3CR1-GFP mice alongside CD11b (Figures 9E–H). No triple-labeled cells were observed, nor were double-labeled cells for CD11b and Iba1. However, all Iba1-positive microglia were also CX3CR1-positive, and there was a distinct population of fractalkine receptor-expressing cells which did not express Iba1. This particular finding is in keeping with that previously reported (Brett et al., 2022). These results further substantiate the notion that extensive MGC heterogeneity exists within the LCIC during its early postnatal critical period.

## Discussion

Results of the present study suggest complement cascade involvement and microglial heterogeneity in the LCIC during its established developmental critical period. C3 and CD11b are transiently expressed as LCIC compartments emerge (Dillingham et al., 2017; Gay et al., 2018; Stinson et al., 2021) and multimodal LCIC afferents segregate accordingly (Lamb-Echegaray et al., 2019; Weakley et al., 2022). Clustering of CD11b-positive MGCs within the modules first and then the matrix, coupled with the sequential disappearance of C3 from said zones, implicate C3-CR3/CD11b signaling as being important for pruning early LCIC multimodal maps. Evidence of heterogenous MGC subpopulations with changing spatial patterns suggests potentially distinct roles and responsibilities for each during early ontogeny. Further classification of these and likely other yet to be identified MGC subsets will be important for providing insights into the mechanisms underlying multisensory circuit assembly, and how aberrant network refinement correlates with certain neurodevelopmental conditions, like ASD and schizophrenia.

### Complement signaling and compartmental pruning within the developing LCIC

**FIGURE 10:**
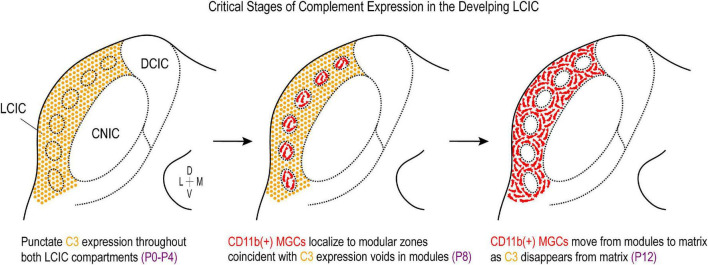
Summary schematic of complement cascade expression in the developing lateral cortex of the inferior colliculus (LCIC). Complement component 3 (C3) expression (orange) is prominent throughout both LCIC compartments at the earliest timepoints examined (P0–P4). Integrin αM (CD11b)-positive microglia (red) are present in the LCIC at these stages (not shown), although do not exhibit any compartmental bias yet. At the critical period peak (P8), C3 expression is selectively lost from modular zones which are now occupied by CD11b-positive microglial cells (MGCs). By P12, CD11b-expressing microglia now occupy the encompassing matrix, where C3 expression is no longer apparent. This developmental sequence implicates complement cascade signaling as a potential key player in compartmental-specific pruning in the nascent LCIC.

The temporal and spatial shaping of auditory and somatosensory afferent patterns correlate not only with emerging LCIC compartments, but also with the discretely-organized complement component protein expression reported here. Both C3 and CD11b are transiently expressed or cleared shortly after the LCIC critical period closure. CD11b-positive MGC occupancy that parallels the disappearance of C3 first within the modules followed by the matrix (Figure 10), speaks toward the likelihood of complement-dependent LCIC pruning that follows a compartmental-specific progression. Analogous C3 experiments to those performed here in CR3KO mice are needed to further substantiate this claim. If indeed C3-CR3 binding is required for selective LCIC pruning, one might hypothesize the observed sequence of C3 loss would not be seen in mutants with compromised signaling. Additional studies that assess relative MGC engulfment activity (e.g., C3-tagged somatosensory and/or auditory terminals) in various mutant strains at critical developmental stages should provide further insights concerning the specific roles microglia play in aspects of LCIC multisensory circuitry refinement.

Synaptic elimination in other systems appears to be mediated in part by other identified opsonins that may also figure prominently in the refinement of early LCIC networks. Externalized phosphatidylserine (PS) induces phagocytic MGC activity through several of its cognate receptors (e.g., TREM2, GPR56; Li et al., 2020; Scott-Hewitt et al., 2020; Raiders et al., 2021), and also partners with upstream complement components such as C1q to instruct engulfment behaviors (Païdassi et al., 2008; Martin et al., 2012). PS localization in the developing hippocampus and visual thalamus is predominantly presynaptic and is selectively targeted by MGCs during established periods of peak pruning. Similar to C3/CR3 knockouts (Schafer et al., 2012), retinogeniculate connections in C1q mutants are insufficiently pruned and exhibit elevated levels of PS and reduced engulfment of PS-labeled material (Scott-Hewitt et al., 2020). Whether PS and/or C1q are present in the developing LCIC, and whether or not each function independently or may influence other aspects of complement signaling within this structure remains to be determined.

### MGC heterogeneity in the nascent LCIC

**FIGURE 11:**
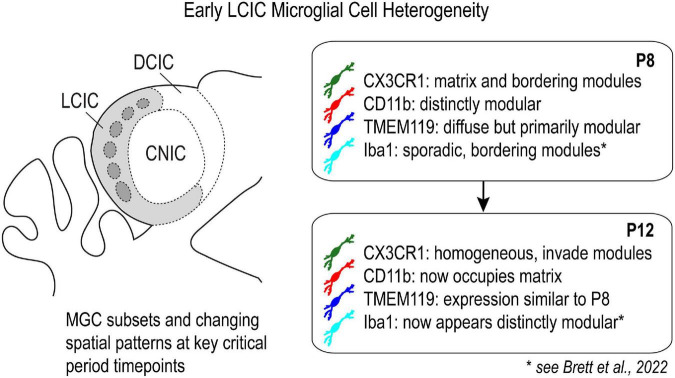
Schematic highlighting differential expression of established microglial cell (MGC) markers in the lateral cortex of the inferior colliculus (LCIC) at key critical period timepoints.

Multiple LCIC microglial subsets with seemingly unique molecular signatures were demonstrated in the present study. Recent single-cell RNA-seq studies in other brain areas reveal distinct MGC transcriptional clusters across the lifespan and in response to injury/inflammation, with the most marked diversity observed during early critical periods of development (De Biase et al., 2017; Hammond et al., 2019; Li et al., 2019; Masuda et al., 2019, 2020; Tan et al., 2020). The significant lack of co-localization for most of the microglial markers examined here, with any observed overlap being the exception rather than the norm, supports the notion of considerable MGC heterogeneity in the developing LCIC (Figure 11). Certainly, there are numerous other established MGC markers (e.g., P2YR12, SIRPα, CD115, etc.; Jurga et al., 2020; Raiders et al., 2021) not explored here that could shed further light on the extent of LCIC MGC heterogeneity. Correlating such immunostaining findings with future RNA-sequencing experiments focused on the nascent LCIC should prove highly informative about the extent of distinct MGC subpopulations, and perhaps the potential responsibilities each have for orchestrating various developmental events.

### MGC pruning and neurodevelopmental conditions

In addition to specific opsonins marking underutilized contacts and debris for selective phagocytosis, other tags such as CD47 appear to serve an alternate role, one of protecting connections deemed necessary from potential elimination (Lehrman et al., 2018; Raiders et al., 2021). A balance of “eat me” and “don’t eat me” tags is important for ensuring that MGC mediated pruning is sufficient and not overstated in the developing brain. Such under- or over-pruning during especially critical windows are thought to correlate with common behavioral phenotypes associated with certain neurodevelopmental conditions. Autism spectrum disorders are typically associated with under-pruning and increased dendritic spine densities compared to neurotypical controls (Hustler and Zhang, 2010; Faust et al., 2021). Several ASD-linked genes are known to influence synaptic pruning in mice, with reported decreases in network refinement and altered social behaviors (Voineagu et al., 2011; De Rubeis et al., 2014; Bourgeron, 2015). In contrast to ASD, various models of schizophrenia consistently point toward over-pruning defects, with reduced spine densities in various brain regions (Fromer et al., 2014; Presumey et al., 2017; Johnson and Stevens, 2018; Yilmaz et al., 2021).

Construction and appropriate sculpting of integrative multisensory circuits during development are likely key for realizing early social-communication milestones. Deficits in certain sensory tasks are highly predictive of later onset and severity of cognitive and behavioral symptoms associated with ASD (Brandwein et al., 2015; Robertson and Baron-Cohen, 2017). In addition to unisensory assessments, impairments in multisensory perceptual binding (Kwakye et al., 2011; Collignon et al., 2013; Stevenson et al., 2014a,b,c, 2018) provide perhaps the most relevant diagnostic criteria, as hallmark features of autism (e.g., difficulties with speech, communication, and social interactions) are inherently multisensory processes. The LCIC with its segregated multimodal afferent-efferent systems likely serves as an important staging ground for further sensory integration at next-level structures (Lesicko et al., 2020). In addition, the LCIC figures prominently in the processing of certain reflexive behaviors (e.g., pre-pulse inhibition, PPI, of the acoustic startle response, ASR), and lesioning of the LCIC is known to significantly alter such responses (Groves et al., 1974; Leitner and Cohen, 1985; Parham and Willott, 1990). Changes in LCIC-mediated response behaviors (ASR, PPI) have been reported extensively in individuals with autism and schizophrenia (Kohl et al., 2013; Haß et al., 2017; Takahashi and Kamio, 2018). Further inquiries that build upon our present findings are needed to determine the extent to which developmental pruning programs may go awry in multisensory structures like the LCIC, and the behavioral consequences that manifest as a result in certain neurodevelopmental conditions.

## Concluding remarks

This study implicates microglia and complement cascade signaling in the compartmental-specific pruning of early connections within the LCIC. Furthermore, our data suggest significant MGC heterogeneity in multisensory regions of the developing midbrain. The mechanisms that govern the precise expression of its molecular tags (e.g. activity-dependent C3/CD47 expression) and the manner in which specific MGC subpopulations are signaled in the LCIC, however, remain largely unaddressed. Careful mapping of the described regional changes and heterogeneities to future single-cell RNA sequencing and spatial transcriptomic studies of LCIC glial populations should deepen our appreciation for the complexity of multisensory midbrain pruning programs and how each may figure into the pathogenesis of certain neurodevelopmental disorders.

## Data availability statement

The raw data supporting the conclusions of this article will be made available by the authors, without undue reservation.

## Ethics statement

The animal study was reviewed and approved by the James Madison University’s Animal Care and Use Committee (approval number: 20-1421).

## Author contributions

JC, SH, and MG contributed to the presented experiments. JC and SH performed the all tissue processing, associated imaging, and data sampling. JC and MG performed the data quantification. MG prepared the all figures and wrote the manuscript. All authors contributed to the article and approved the submitted version.

## Footnotes

1
www.clarechemical.com

2
https://www.transnetyx.com/

3
https://imagej.net/
